# Monitoring Progress in Population Health: Trends in Premature Death Rates

**DOI:** 10.5888/pcd10.130210

**Published:** 2013-12-26

**Authors:** Patrick L. Remington, Bridget B. Catlin, David A. Kindig

**Affiliations:** Author Affiliations: Bridget B. Catlin, David A. Kindig, Population Health Institute, Department of Population Health Sciences, University of Wisconsin School of Medicine and Public Health, Madison, Wisconsin.

## Abstract

**Introduction:**

Trends in population health outcomes can be monitored to evaluate the performance of population health systems at the national, state, and local levels. The objective of this study was to compare and contrast 4 measures for assessing progress in population health improvement by using age-adjusted premature death rates as a summary measure of the overall health outcomes in the United States and in all 50 states.

**Methods:**

To evaluate the performance of statewide population health systems during the past 20 years, we used 4 measures of age-adjusted premature (<75 years of age) death rates: current rates (2009), baseline trends (1990s), follow-up trends (2000s), and changes in trends from baseline to the follow-up periods (ie, “bending the curve”).

**Results:**

Current premature death rates varied by approximately twofold, with the lowest rate in Minnesota (268 deaths per 100,000) and the highest rate in Mississippi (482 deaths per 100,000). Rates improved the most in New York during the baseline period (−3.05% per year) and in New Jersey during the follow-up period (−2.87% per year), whereas Oklahoma ranked last in trends during both periods (−0.30%/y, baseline; +0.18%/y, follow-up). Trends improved the most in Connecticut, bending the curve downward by −1.03%; trends worsened the most in New Mexico, bending the curve upward by 1.21%.

**Discussion:**

Current premature death rates, recent trends, and changes in trends vary by state in the United States. Policy makers can use these measures to evaluate the long-term population health impact of broad health care, behavioral, social, and economic investments in population health.

## Introduction

Interest in measuring and monitoring the health of populations has grown during the past 2 decades. As researchers improve ways to measure health, results show significant differences in the health of entire populations (1,2). The ranking of national health system performance in 2000 listed the United States at 37th in the world and led to discussion and criticism about the methods used to measure health (3–5). For more than 20 years, America’s Health Rankings has ranked the health of all 50 states by using a composite measure including health outcomes and determinants (6). A population health model was developed in Wisconsin in 2003 to rank the health of its 72 counties, and since 2010, this model has been used to rank the health of each county in all 50 states (2,7).

Despite the interest in these and other health rankings, other methods may be more useful for evaluating the impact of efforts to improve public health. For example, trends in health outcomes and health factors can be monitored to evaluate past progress and set goals for the future. The objective of this study was to compare and contrast 4 measures for assessing progress in population health improvement by using age-adjusted premature death rates as a summary measure of the overall health outcomes in the United States and in all 50 states (8).

## Methods

Death data from 1990 to 2009 were obtained from the Centers for Disease Control and Prevention website Wide-ranging Online Data for Epidemiologic Research (WONDER) (9). We used age-adjusted death rates for people younger than 75 as an indicator of premature mortality. We chose this indicator for our analysis because deaths for those younger than 75 are more amenable to prevention, and unlike life expectancy and years of person-lives lost, this measure is easy to calculate and a straightforward, sensitive indicator of differences in population health (10,11). These rates were obtained for each year and age-adjusted to the US 2000 population. We calculated 4 measures for the United States and for each state: current premature death rates, baseline trends in premature death rates, follow-up trends in premature death rates, and changes in trends in premature death rates (ie, “bending the curve”):

- **Current premature death rates.** The age-adjusted premature death rate for 2009.
- **Trends in baseline and follow-up premature death rates. **Premature death rate trends were determined for 2 periods: 1990s (1990–1999) and 2000s (2000–2009). To compare trends, the first period (1990–1999) was considered the baseline period, and the second period (2000–2009) was considered the follow-up period. Trends were calculated using the estimated annual percentage change in age-adjusted premature death rates for each period. The annual percentage change is the average rate of change per year and assumes that the rate of change over time is constant (ie, it is based on the slope of the natural log of the rates). The value is given as a percentage, such as an approximate 1% per year decrease, and is a standard way of measuring changes in population rates (12). A negative value reflects decreasing premature death rates (ie, improvement), whereas a positive value reflects increasing premature death rates (ie, worsening).
- **Changes in trends in premature death rates (ie, bending the curve).** “Bending the curve” is defined as the difference between the follow-up trends (2000s) and baseline trends (1990s) in premature death rates. A negative value reflects an improvement in trend from baseline to follow-up (bending the curve downward), whereas a positive value reflects less improvement in the follow-up period than during the baseline period (bending the curve upward). We also plotted each state’s baseline and follow-up trends to show states that have improving and worsening trends.

Trends in rates (annual percentage changes) were calculated by using SPSS version 19 (IBM Corporation, Armonk, New York). We analyzed data by state and by US census region (West, Midwest, Northeast, and South).

## Results

**Figure 1:**
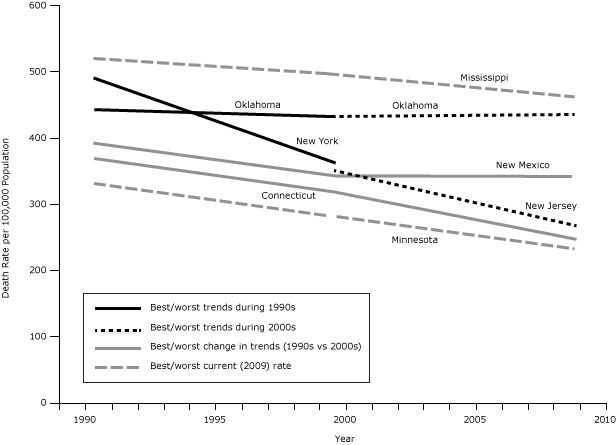
Trends in premature (<75 y, age-adjusted) death rates from 1990 to 2009 for the best- and worst-ranked states, based on current (2009) death rates; follow-up (2000s) trends; and changes in trends from the baseline (1990s) to the follow-up (2000s) period. Data for this graph are available in the Table.

In the United States in 2009, the age-adjusted premature death rate was 346 deaths per 100,000 (Table). Rates varied approximately twofold across the United States: the lowest rate was in Minnesota (268 deaths per 100,000 [Figure 1]), followed by Connecticut (276), Vermont (278), New Hampshire (281), and Massachusetts (283); the highest rate was in Mississippi (482 deaths per 100,000 [Figure 1]), followed by West Virginia (464), Alabama (463), Louisiana (456), and Oklahoma (452). States with the lowest rates were in the Northeast, Midwest, and West, and states with the highest rates were in the South.

**Table:** Trends in Age-Adjusted Premature (<75 Years of Age) Death Rates in the United States, 1990–2009

State	Baseline Trend (1990–1999)		Follow-up Trend (2000–2009)		Change in Trend (1990s to 2000s)		Current Rate (2009)	
	APC, %	Rank	APC, %	Rank	Change in APC,^a^ %	Rank	Rate^b^	Rank
Alabama	−0.80	40	−0.34	45	0.46	42	463	48
Alaska	−1.69	6	−1.05	36	0.64	46	351	32
Arizona	−1.29	23	−1.79	15	−0.49	10	326	22
Arkansas	−0.80	40	−0.27	46	0.53	44	449	45
California	−2.18	2	−2.06	8	0.11	33	289	8
Colorado	−1.39	18	−1.65	17	−0.26	22	297	12
Connecticut	−1.49	13	−2.51	4	−1.03	1	276	2
Delaware	−1.59	11	−1.34	29	0.25	38	355	33
Florida	−1.49	13	−1.16	32	0.33	41	341	25
Georgia	−1.49	13	−2.17	7	−0.68	7	388	41
Hawaii	−1.00	35	−0.27	47	0.72	48	288	7
Idaho	−1.00	35	−1.36	28	−0.36	17	309	16
Illinois	−1.69	6	−2.18	6	−0.50	9	337	24
Indiana	−0.80	40	−1.19	31	−0.40	15	381	39
Iowa	−1.00	35	−0.86	39	0.14	34	312	17
Kansas	−0.60	47	−0.89	38	−0.30	18	345	27
Kentucky	−0.90	38	−0.61	42	0.28	40	449	44
Louisiana	−1.09	29	−0.90	37	0.20	36	456	47
Maine	−1.39	18	−1.43	26	−0.04	31	314	18
Maryland	−1.39	18	−2.27	5	−0.88	3	342	26
Massachusetts	−2.08	4	−2.56	3	−0.48	12	283	5
Michigan	−1.19	26	−1.59	20	−0.40	14	360	34
Minnesota	−1.49	13	−1.93	11	−0.44	13	268	1
Mississippi	−0.50	49	−0.68	40	−0.18	26	482	50
Missouri	−0.70	44	−1.07	35	−0.37	16	384	40
Montana	−1.19	26	−0.62	41	0.57	45	347	29
Nebraska	−1.09	29	−1.28	30	−0.19	25	304	14
Nevada	−1.39	18	−1.48	23	−0.09	29	370	36
New Hampshire	−1.69	6	−1.85	14	−0.16	28	281	4
New Jersey	−2.18	2	−2.87	1	−0.69	6	291	9
New Mexico	−1.29	23	−0.08	48	1.21	50	362	35
New York	−3.05	1	−2.79	2	0.26	39	296	10
North Carolina	−1.29	23	−1.57	22	−0.28	19	372	37
North Dakota	−1.09	29	−0.45	44	0.64	47	317	19
Ohio	−1.09	29	−1.11	33	−0.02	32	378	38
Oklahoma	-0.30	50	0.18	50	0.48	43	452	46
Oregon	−1.09	29	−1.64	18	−0.55	8	318	21
Pennsylvania	−1.39	18	−1.45	25	−0.06	30	349	30
Rhode Island	−1.69	6	−1.87	12	−0.19	24	306	15
South Carolina	−1.19	26	−1.47	24	−0.28	20	407	42
South Dakota	−0.80	40	−1.61	19	−0.82	4	318	20
Tennessee	−0.60	47	−1.08	34	−0.49	11	432	43
Texas	−1.59	11	−1.43	27	0.16	35	351	31
Utah	−0.70	44	−1.59	21	−0.89	2	285	6
Vermont	−1.88	5	−2.05	9	−0.16	27	278	3
Virginia	−1.69	6	−1.95	10	−0.27	21	330	23
Washington	−1.49	13	−1.71	16	−0.22	23	302	13
West Virginia	−0.90	38	0.17	49	1.07	49	464	49
Wisconsin	−1.09	29	−1.85	13	−0.76	5	296	10
Wyoming	−0.70	44	−0.47	43	0.23	37	347	28
United States	−1.49	—	−1.59	—	−0.10	—	346	—

Abbreviations: APC, annual percentage change.

a Change in APC from the 1990s to the 2000s.

b Age-adjusted premature (<75 y) death rate (per 100,000).

Premature death rates declined during the 1990s in the United States at an annual rate of change of −1.49% per year (Table). All states improved during the 1990s; the greatest rate of improvement was in New York (−3.05% per year [Figure 1]), followed by California and New Jersey (both −2.18% per year), Massachusetts (−2.08% per year), and Vermont (−1.88% per year); the slowest rate of improvement was in Oklahoma (−0.30% per year [Figure 1]), followed by Mississippi (−0.50% per year), and Tennessee and Kansas (both −0.60% per year). States with the greatest rates of improvement were in the Northeast, and states with the least improvement were mostly in the South and Midwest.

Premature death rates continued to decline in the United States during the 2000s, declining by −1.59% per year (Table). Again, trends varied by state, with the greatest improvement in New Jersey (−2.87% per year [Figure 1]), followed by New York (−2.79% per year), Massachusetts (−2.56% per year), Connecticut (−2.51% per year), and Maryland (−2.27% per year). The rates in 2 states increased during this time: Oklahoma (+0.18% per year [Figure 1]) and West Virginia (+0.17% per year); rates decreased only slightly in New Mexico (−0.08% per year), and Hawaii and Arkansas (both −0.27% per year). States with the greatest improvement continued to be in the Northeast, Midwest, and West; and states with the least improvement (or increases in death rates) were generally located in the South

**Figure 2:**
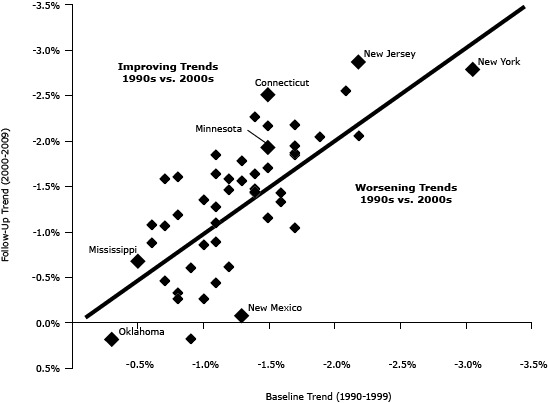
Correlation between baseline and follow-up trends for each state. States in which trends improved are plotted above the line, and states in which trends worsened are plotted below the line. Each state is represented by a diamond. The states with the best/worst performance are identified. Data for this graph are available in the Table. Data source: Centers for Disease Control and Prevention’s Wide-ranging Online Data for Epidemiologic Research (WONDER).

In the United States, the annual percentage change in death rates during the 2000s was only slightly greater than during the 1990s — representing improvement of−0.10% per year. Thirty-two states had faster rates of improvement during the 2000s than during the 1990s, and 18 states had a decline in the rate of improvement (Table and Figure 2). 

We found the greatest improvement in trend (−1.03%) in Connecticut (from −1.49% during the 1990s to −2.51% per year during the 2000s [Figure 2]), followed by Utah (−0.89%), Maryland (−0.88%), South Dakota (−0.82%), and Wisconsin (−0.76%). In contrast, the worst change in trend (+1.21%) was in New Mexico (from −1.29% during the 1990s to −0.08% per year during the 2000s [Figure 2]), followed by West Virginia (+1.07%), Hawaii (+0.72%), North Dakota (+0.64%), and Alaska (+0.64%). States with the greatest improvement in trend were from all regions of the United States, whereas states that had declining trends in the 2000s were mostly located in the South.

## Discussion

Our study demonstrates improvements in the overall health of the nation during the past 2 decades: the age-adjusted premature death rates declined by about 1.5% per year during each decade. However, trends varied from state to state; some states, such as New York, had consistent and remarkable declines that were 2 or 3 times greater than the declines in the United States overall, and other states, such as Oklahoma, had no improvement or even a slight increase in premature death rates during this period. Our study also shows that trends were not consistent for some states during this time; states such as Connecticut improved in premature death rate trends and other states, such as New Mexico, had less progress.

Each measure used in our study served a different purpose for monitoring population health performance. First, the use of the *current* rate of premature death showed remarkable differences in health across the United States. Premature death rates were approximately 2 times greater in Mississippi (ranked 50th) than in Minnesota (ranked 1st). Reporting how a place ranks on current death rates — as is done in the America’s Health Rankings and County Health Rankings — stimulates interest among the news media and engages community and state leaders, especially in states or counties that are at the top or bottom of such a list (6,7,13,14). Such an absolute level of achievement in health is an important way of measuring population health, because the highest ranking places can serve as exemplars to those ranking lower and can stimulate action and investment for improvement. On the other hand, rankings based on current data do not reflect progress that may have been made, especially for states ranking at the bottom of the list.

Second, measuring *trends* in population health outcomes enables communities to evaluate the differential impact of changes in health determinants and offers all communities — regardless of their overall health — the ability to see improvement. The rankings of trends — both in the 1990s and 2000s — are different from the rankings of the current premature death rates. For example, New Jersey leads the nation in the rate of improvement during the past decade, and Oklahoma is last, having seen almost no change. These 2 states had similar premature death rates in 1990, whereas 2 decades later, New Jersey’s premature death rate is now almost 40% lower than Oklahoma’s. This dramatic difference in progress should prompt researchers and policy makers to explore the reasons and possible strategies to close this gap.

Finally, measuring *changes in trends* over time — or bending the curve — may be most useful for evaluating the impact of broad health care, behavioral, social, and economic changes on trends in population health over long periods of time. This is a common approach for evaluating the impact of interventions because it controls for baseline differences in the historical trends between communities. This approach has been used for describing changes in the rates of increase in health care costs (15). Program interventions or policy changes are likely to influence the health of populations — including rates of premature death — several years later. The ranking of states using this measure produces yet another set of high-performing states, ranging from the best change in trends in Connecticut to the worst in New Mexico, one of 18 states whose progress during the 1990s slowed during the 2000s. Measuring changes in trends over time — bending the population health curve — may help us to better understand how changes in the multiple determinants of health during the 1990s have affected health outcomes during the ensuing decade and to identify the best measure of the effectiveness of broad-based efforts to improve public health.

Our study has several limitations. First, although ranking is useful for contrasting differences between state rates and trends, differences between states that are closely ranked may be statistically insignificant. Further analyses should be conducted to determine the reasons for differences in trends, such as differences in cause-specific death rates, trends in other health determinants, or effects of changing demographics.

Monitoring progress in the health of populations will become increasingly important as the US health care system moves toward rewarding improvement in health outcomes. Because many public health interventions are implemented at the local level, the impact of these interventions should also be evaluated in smaller populations. However, in smaller populations such as counties or cities, both current and trends in premature death rates — and other measures of health outcomes — will have more statistical variability. Combining several years of data improves reliability but limits the potential to monitor trends over time. In addition, measures that are derived from surveys, such as telephone surveys of health behaviors (16), have large confidence intervals and are often too imprecise to enable evaluation of changes over time. Other measures may be available for the entire population (eg, census measures, rates of low birth weight) and may provide more reliable estimates (17). For example, smoking rates among pregnant women are available for all births over time and can be used to monitor trends in states, counties, or smaller communities (18).

States and communities throughout the nation are striving to improve the health of the public, not only by improving health care quality and access but also by implementing public health and environmental policies and programs and investing in educational systems and economic development efforts (19). Trends in population health outcomes — such as premature death rates — can be used to evaluate the long-term population health impact of these changes, stimulate discussion and action, and ensure that the nation achieves the goal of longer, healthier lives for all.
